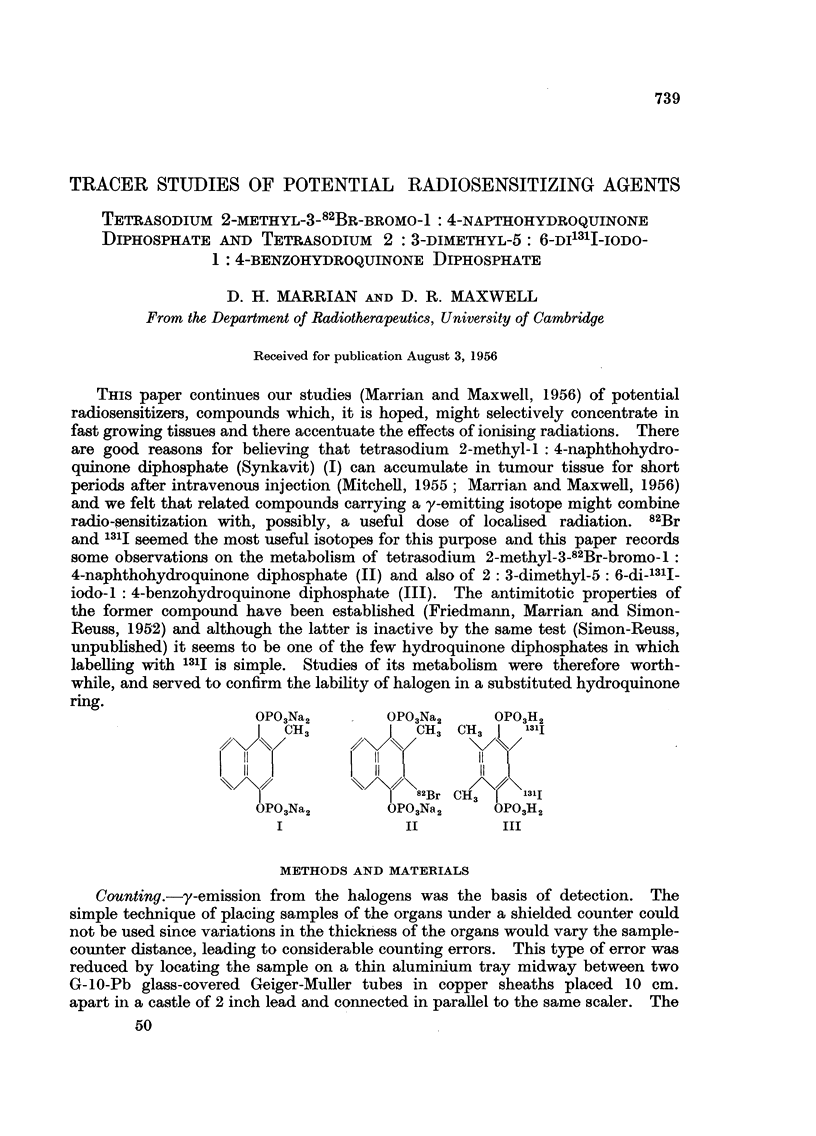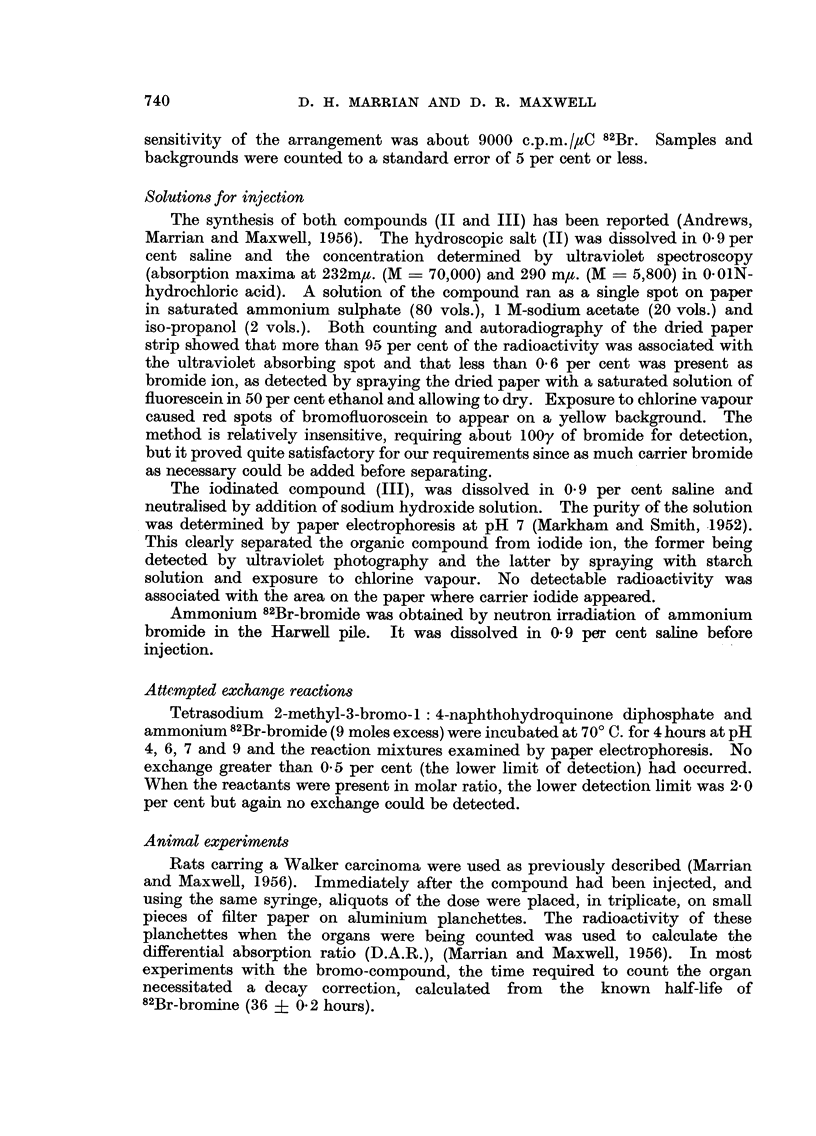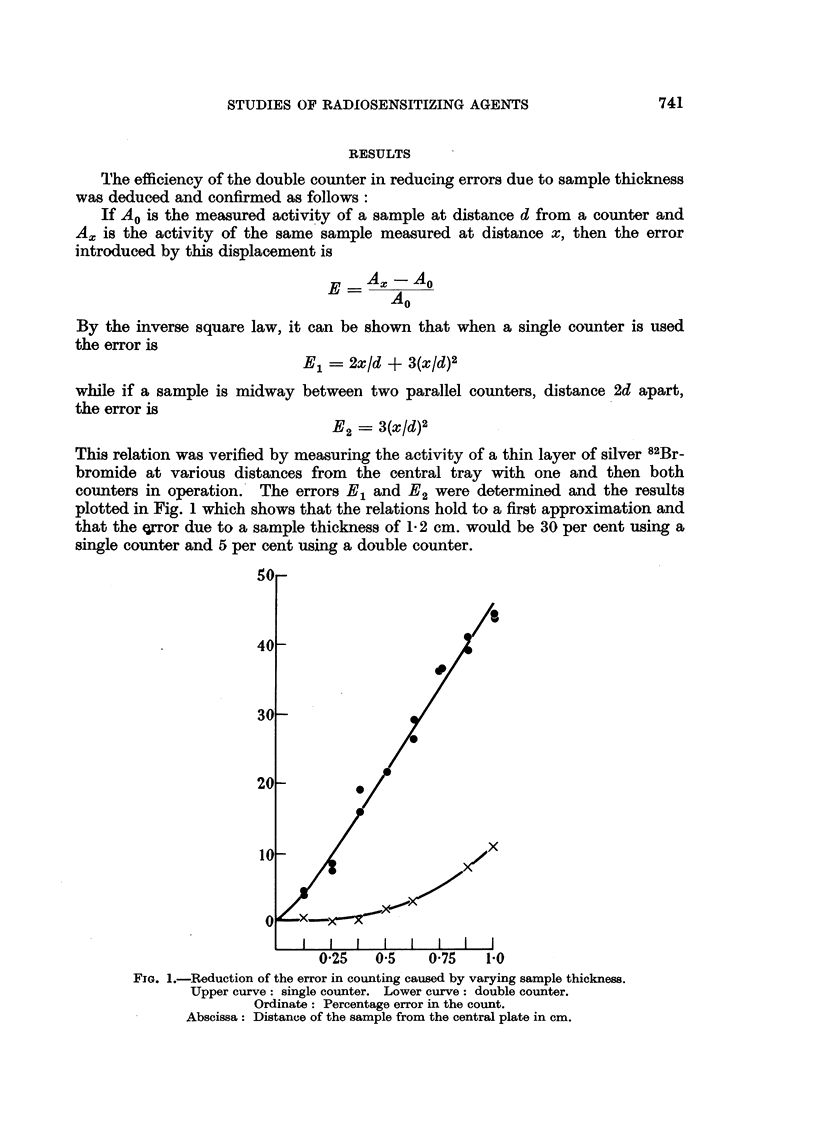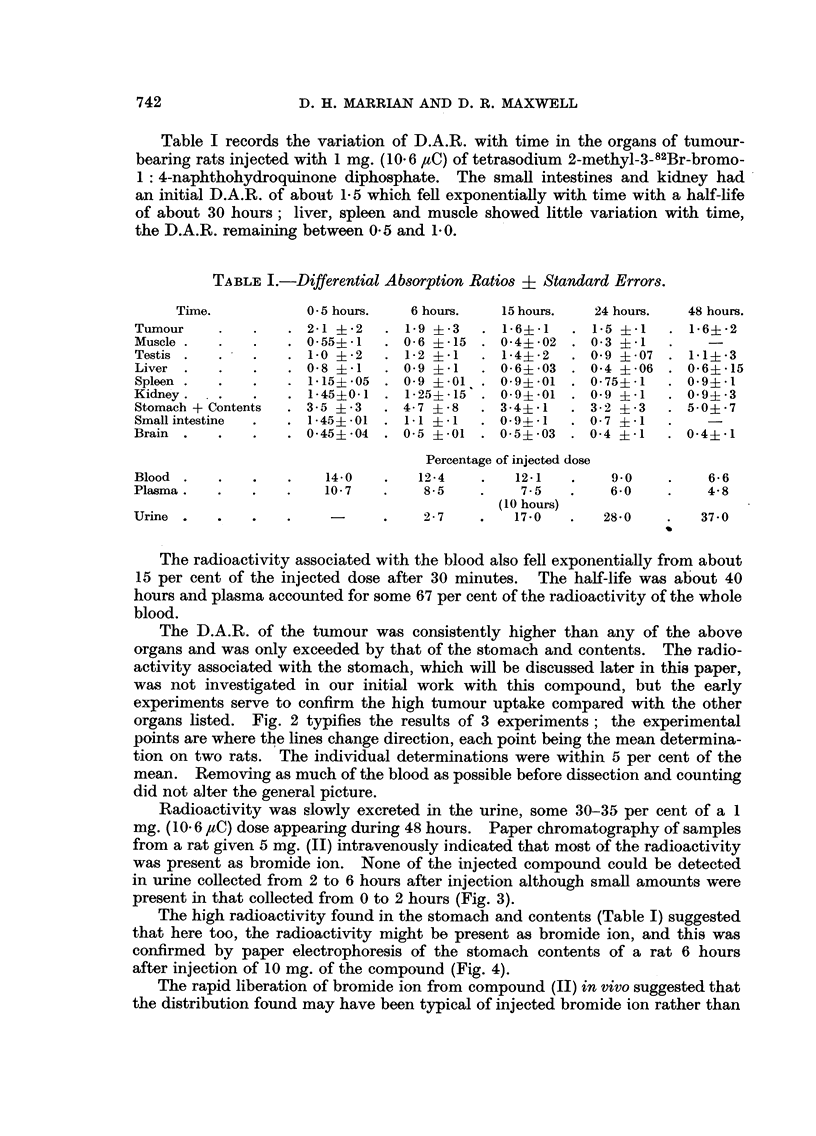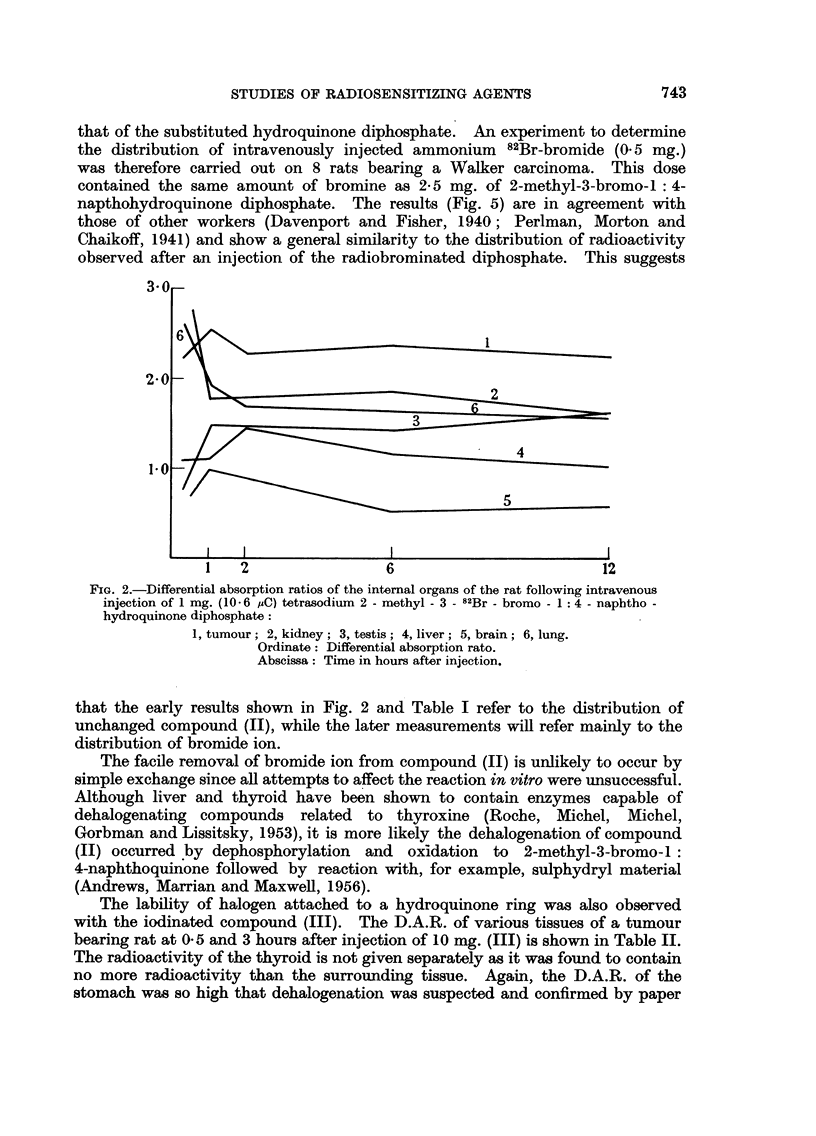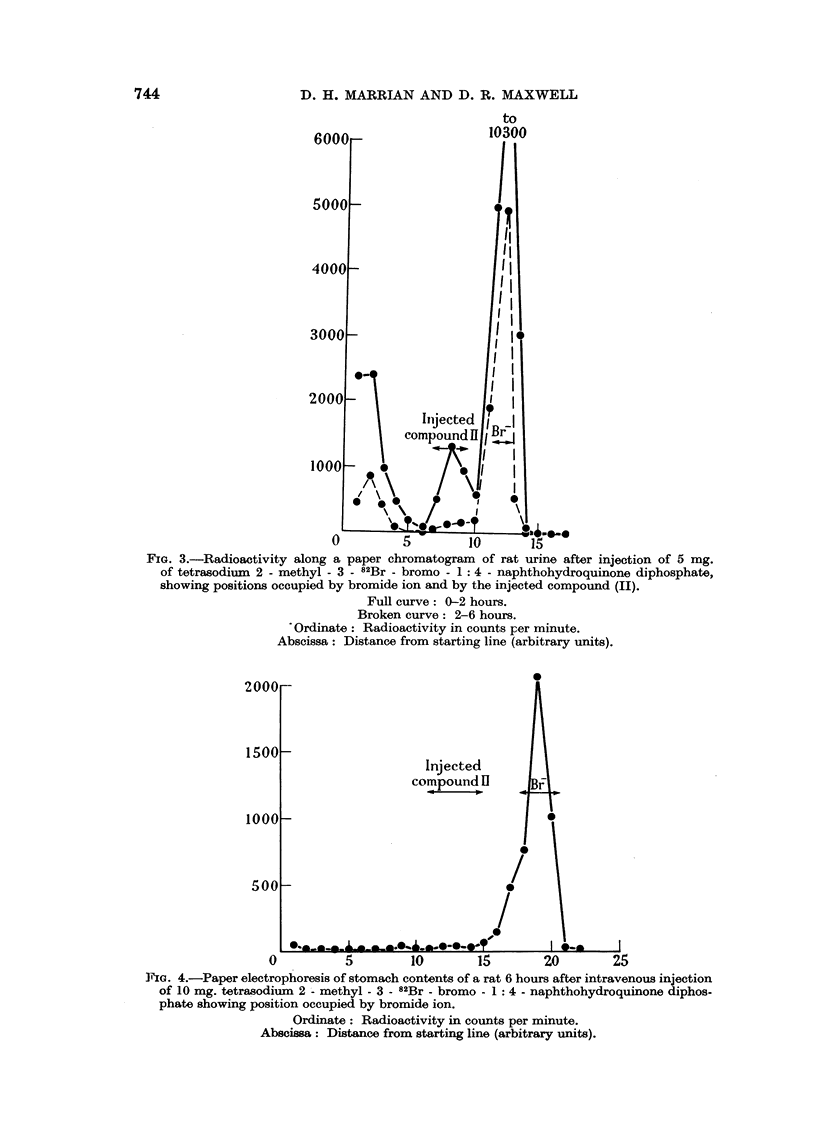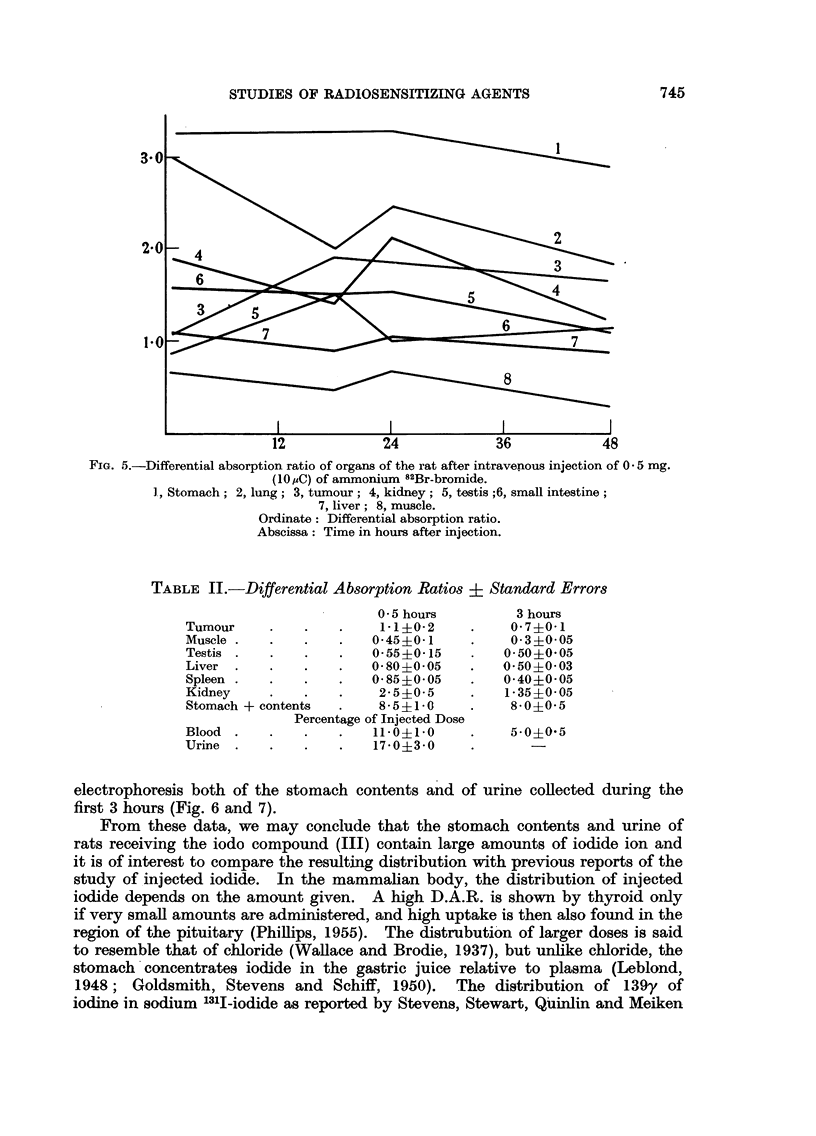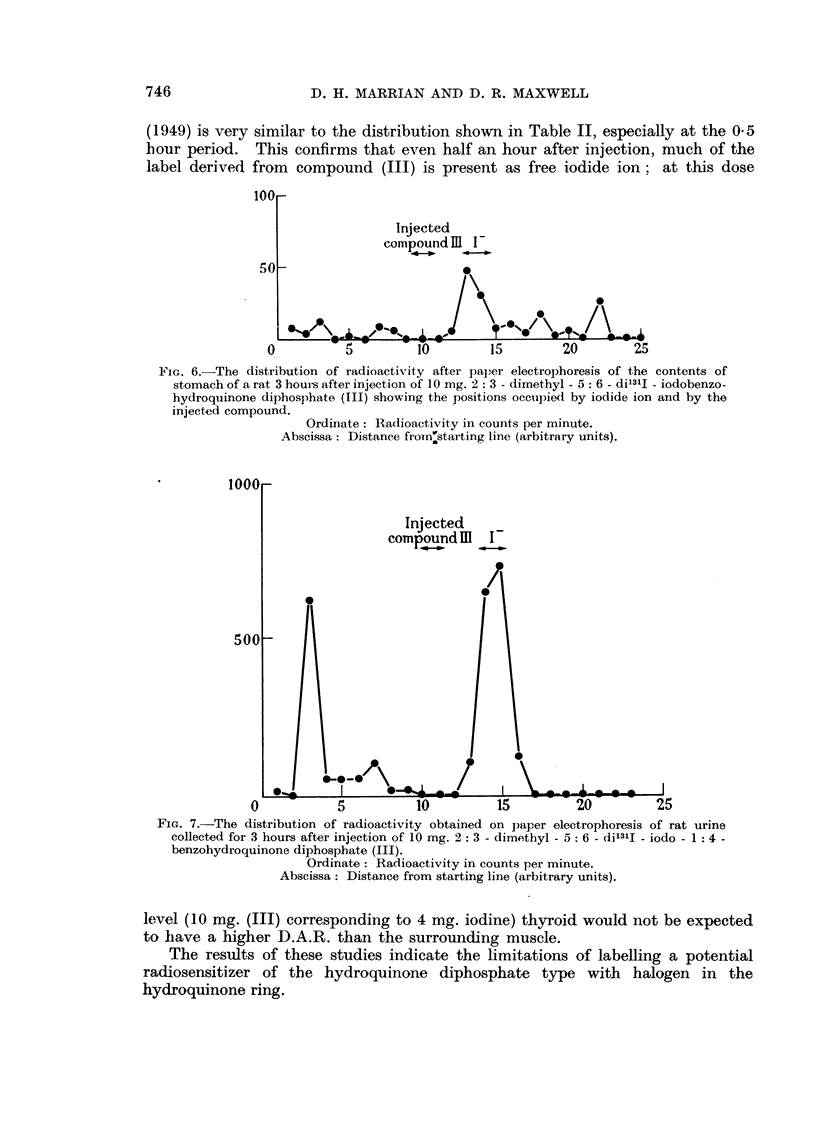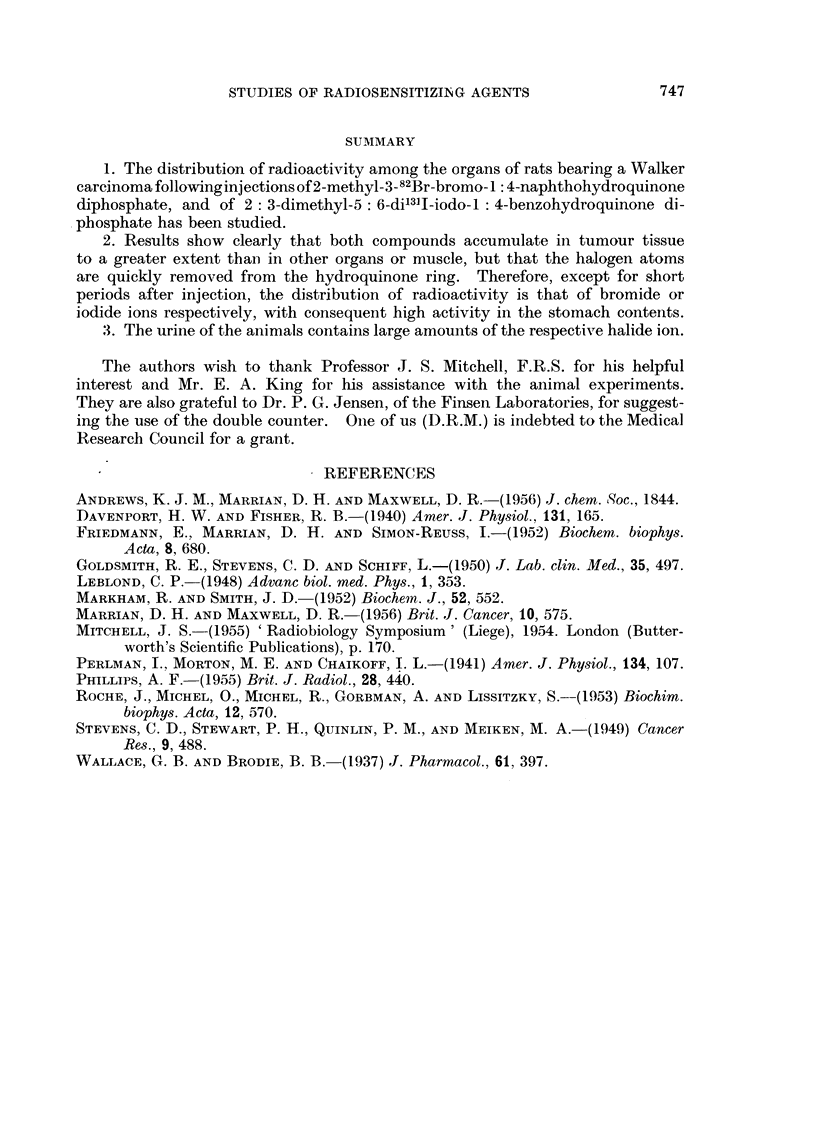# Tracer Studies of Potential Radiosensitizing Agents

**DOI:** 10.1038/bjc.1956.90

**Published:** 1956-12

**Authors:** D. H. Marrian, D. R. Maxwell


### TRACER STUDIES OF POTENTIAL RADIOSENSITIZING AGENTS

### TETRASODIUM 2-METHYL-3-$^{82}$BR-BROMO-1 : 4-NAPTHOHYDROQUINONE DIPHOSPHATE AND TETRASODIUM 2 : 3-DIMETHYL-5 : 6-DI$^{131}$I-IODO-1 : 4-BENZOHYDROQUINONE DIPHOSPHATE

D. H. MARRIAN AND D. R. MAXWELL

*From the Department of Radiotherapeutics, University of Cambridge*

Received for publication August 3, 1956

THIS paper continues our studies (Marrian and Maxwell, 1956) of potential radiosensitizers, compounds which, it is hoped, might selectively concentrate in fast growing tissues and there accentuate the effects of ionising radiations. There are good reasons for believing that tetrasodium 2-methyl-1 : 4-naphthohydroquinone diphosphate (Synkavit) (I) can accumulate in tumour tissue for short periods after intravenous injection (Mitchell, 1955 ; Marrian and Maxwell, 1956) and we felt that related compounds carrying a $\gamma$-emitting isotope might combine radio-sensitization with, possibly, a useful dose of localised radiation. $^{82}$Br and $^{131}$I seemed the most useful isotopes for this purpose and this paper records some observations on the metabolism of tetrasodium 2-methyl-3-$^{82}$Br-bromo-1 : 4-naphthohydroquinone diphosphate (II) and also of 2 : 3-dimethyl-5 : 6-di-$^{131}$I-iodo-1 : 4-benzohydroquinone diphosphate (III). The antimitotic properties of the former compound have been established (Friedmann, Marrian and Simon-Reuss, 1952) and although the latter is inactive by the same test (Simon-Reuss, unpublished) it seems to be one of the few hydroquinone diphosphates in which labelling with $^{131}$I is simple. Studies of its metabolism were therefore worthwhile, and served to confirm the lability of halogen in a substituted hydroquinone ring.

I II III

### METHODS AND MATERIALS

*Counting.*—$\gamma$-emission from the halogens was the basis of detection. The simple technique of placing samples of the organs under a shielded counter could not be used since variations in the thickness of the organs would vary the sample-counter distance, leading to considerable counting errors. This type of error was reduced by locating the sample on a thin aluminium tray midway between two G-10-Pb glass-covered Geiger-Muller tubes in copper sheaths placed 10 cm. apart in a castle of 2 inch lead and connected in parallel to the same scaler. The

50

sensitivity of the arrangement was about 9000 c.p.m./$\mu$C $^{82}$Br. Samples and backgrounds were counted to a standard error of 5 per cent or less.

*Solutions for injection*

The synthesis of both compounds (II and III) has been reported (Andrews, Marrian and Maxwell, 1956). The hydroscopic salt (II) was dissolved in 0·9 per cent saline and the concentration determined by ultraviolet spectroscopy (absorption maxima at 232m$\mu$. (M = 70,000) and 290 m$\mu$. (M = 5,800) in 0·01N-hydrochloric acid). A solution of the compound ran as a single spot on paper in saturated ammonium sulphate (80 vols.), 1 M-sodium acetate (20 vols.) and iso-propanol (2 vols.). Both counting and autoradiography of the dried paper strip showed that more than 95 per cent of the radioactivity was associated with the ultraviolet absorbing spot and that less than 0·6 per cent was present as bromide ion, as detected by spraying the dried paper with a saturated solution of fluorescein in 50 per cent ethanol and allowing to dry. Exposure to chlorine vapour caused red spots of bromofluoroscein to appear on a yellow background. The method is relatively insensitive, requiring about 100$\gamma$ of bromide for detection, but it proved quite satisfactory for our requirements since as much carrier bromide as necessary could be added before separating.

The iodinated compound (III), was dissolved in 0·9 per cent saline and neutralised by addition of sodium hydroxide solution. The purity of the solution was determined by paper electrophoresis at pH 7 (Markham and Smith, 1952). This clearly separated the organic compound from iodide ion, the former being detected by ultraviolet photography and the latter by spraying with starch solution and exposure to chlorine vapour. No detectable radioactivity was associated with the area on the paper where carrier iodide appeared.

Ammonium $^{82}$Br-bromide was obtained by neutron irradiation of ammonium bromide in the Harwell pile. It was dissolved in 0·9 per cent saline before injection.

*Attempted exchange reactions*

Tetrasodium 2-methyl-3-bromo-1 : 4-naphthohydroquinone diphosphate and ammonium $^{82}$Br-bromide (9 moles excess) were incubated at 70° C. for 4 hours at pH 4, 6, 7 and 9 and the reaction mixtures examined by paper electrophoresis. No exchange greater than 0·5 per cent (the lower limit of detection) had occurred. When the reactants were present in molar ratio, the lower detection limit was 2·0 per cent but again no exchange could be detected.

*Animal experiments*

Rats carring a Walker carcinoma were used as previously described (Marrian and Maxwell, 1956). Immediately after the compound had been injected, and using the same syringe, aliquots of the dose were placed, in triplicate, on small pieces of filter paper on aluminium planchettes. The radioactivity of these planchettes when the organs were being counted was used to calculate the differential absorption ratio (D.A.R.), (Marrian and Maxwell, 1956). In most experiments with the bromo-compound, the time required to count the organ necessitated a decay correction, calculated from the known half-life of $^{82}$Br-bromine (36 $\pm$ 0·2 hours).

### RESULTS

The efficiency of the double counter in reducing errors due to sample thickness was deduced and confirmed as follows :

If $A_0$ is the measured activity of a sample at distance $d$ from a counter and $A_x$ is the activity of the same sample measured at distance $x$, then the error introduced by this displacement is

$$E = \frac{A_x - A_0}{A_0}$$

By the inverse square law, it can be shown that when a single counter is used the error is

$$E_1 = 2x/d + 3(x/d)^2$$

while if a sample is midway between two parallel counters, distance $2d$ apart, the error is

$$E_2 = 3(x/d)^2$$

This relation was verified by measuring the activity of a thin layer of silver $^{82}$Br-bromide at various distances from the central tray with one and then both counters in operation. The errors $E_1$ and $E_2$ were determined and the results plotted in Fig. 1 which shows that the relations hold to a first approximation and that the error due to a sample thickness of 1·2 cm. would be 30 per cent using a single counter and 5 per cent using a double counter.

FIG. 1.—Reduction of the error in counting caused by varying sample thickness.
Upper curve : single counter. Lower curve : double counter.
Ordinate : Percentage error in the count.
Abscissa : Distance of the sample from the central plate in cm.

Table I records the variation of D.A.R. with time in the organs of tumour-bearing rats injected with 1 mg. (10·6 $\mu$C) of tetrasodium 2-methyl-3-$^{82}$Br-bromo-1 : 4-naphthohydroquinone diphosphate. The small intestines and kidney had an initial D.A.R. of about 1·5 which fell exponentially with time with a half-life of about 30 hours ; liver, spleen and muscle showed little variation with time, the D.A.R. remaining between 0·5 and 1·0.

TABLE I.—*Differential Absorption Ratios ± Standard Errors.*

| Time. | 0·5 hours. | 6 hours. | 15 hours. | 24 hours. | 48 hours. |
|---|---|---|---|---|---|
| Tumour | 2·1 ± ·2 | 1·9 ± ·3 | 1·6 ± ·1 | 1·5 ± ·1 | 1·6 ± ·2 |
| Muscle | 0·55 ± ·1 | 0·6 ± ·15 | 0·4 ± ·02 | 0·3 ± ·1 | — |
| Testis | 1·0 ± ·2 | 1·2 ± ·1 | 1·4 ± ·2 | 0·9 ± ·07 | 1·1 ± ·3 |
| Liver | 0·8 ± ·1 | 0·9 ± ·1 | 0·6 ± ·03 | 0·4 ± ·06 | 0·6 ± ·15 |
| Spleen | 1·15 ± ·05 | 0·9 ± ·01 | 0·9 ± ·01 | 0·75 ± ·1 | 0·9 ± ·1 |
| Kidney | 1·45 ± 0·1 | 1·25 ± ·15 | 0·9 ± ·01 | 0·9 ± ·1 | 0·9 ± ·3 |
| Stomach + Contents | 3·5 ± ·3 | 4·7 ± ·8 | 3·4 ± ·1 | 3·2 ± ·3 | 5·0 ± ·7 |
| Small intestine | 1·45 ± ·01 | 1·1 ± ·1 | 0·9 ± ·1 | 0·7 ± ·1 | — |
| Brain | 0·45 ± ·04 | 0·5 ± ·01 | 0·5 ± ·03 | 0·4 ± ·1 | 0·4 ± ·1 |
| | | | Percentage of injected dose | | |
| Blood | 14·0 | 12·4 | 12·1 | 9·0 | 6·6 |
| Plasma | 10·7 | 8·5 | 7·5 | 6·0 | 4·8 |
| | | | (10 hours) | | |
| Urine | — | 2·7 | 17·0 | 28·0 | 37·0 |

The radioactivity associated with the blood also fell exponentially from about 15 per cent of the injected dose after 30 minutes. The half-life was about 40 hours and plasma accounted for some 67 per cent of the radioactivity of the whole blood.

The D.A.R. of the tumour was consistently higher than any of the above organs and was only exceeded by that of the stomach and contents. The radioactivity associated with the stomach, which will be discussed later in this paper, was not investigated in our initial work with this compound, but the early experiments serve to confirm the high tumour uptake compared with the other organs listed. Fig. 2 typifies the results of 3 experiments ; the experimental points are where the lines change direction, each point being the mean determination on two rats. The individual determinations were within 5 per cent of the mean. Removing as much of the blood as possible before dissection and counting did not alter the general picture.

Radioactivity was slowly excreted in the urine, some 30–35 per cent of a 1 mg. (10·6 $\mu$C) dose appearing during 48 hours. Paper chromatography of samples from a rat given 5 mg. (II) intravenously indicated that most of the radioactivity was present as bromide ion. None of the injected compound could be detected in urine collected from 2 to 6 hours after injection although small amounts were present in that collected from 0 to 2 hours (Fig. 3).

The high radioactivity found in the stomach and contents (Table I) suggested that here too, the radioactivity might be present as bromide ion, and this was confirmed by paper electrophoresis of the stomach contents of a rat 6 hours after injection of 10 mg. of the compound (Fig. 4).

The rapid liberation of bromide ion from compound (II) *in vivo* suggested that the distribution found may have been typical of injected bromide ion rather than that of the substituted hydroquinone diphosphate. An experiment to determine the distribution of intravenously injected ammonium $^{82}$Br-bromide (0·5 mg.) was therefore carried out on 8 rats bearing a Walker carcinoma. This dose contained the same amount of bromine as 2·5 mg. of 2-methyl-3-bromo-1 : 4-napthohydroquinone diphosphate. The results (Fig. 5) are in agreement with those of other workers (Davenport and Fisher, 1940; Perlman, Morton and Chaikoff, 1941) and show a general similarity to the distribution of radioactivity observed after an injection of the radiobrominated diphosphate. This suggests

FIG. 2.—Differential absorption ratios of the internal organs of the rat following intravenous injection of 1 mg. (10·6 $\mu$C) tetrasodium 2 - methyl - 3 - $^{82}$Br - bromo - 1 : 4 - naphtho - hydroquinone diphosphate :

1, tumour ; 2, kidney ; 3, testis ; 4, liver ; 5, brain ; 6, lung.
Ordinate : Differential absorption rato.
Abscissa : Time in hours after injection.

that the early results shown in Fig. 2 and Table I refer to the distribution of unchanged compound (II), while the later measurements will refer mainly to the distribution of bromide ion.

The facile removal of bromide ion from compound (II) is unlikely to occur by simple exchange since all attempts to affect the reaction *in vitro* were unsuccessful. Although liver and thyroid have been shown to contain enzymes capable of dehalogenating compounds related to thyroxine (Roche, Michel, Michel, Gorbman and Lissitsky, 1953), it is more likely the dehalogenation of compound (II) occurred by dephosphorylation and oxidation to 2-methyl-3-bromo-1 : 4-naphthoquinone followed by reaction with, for example, sulphydryl material (Andrews, Marrian and Maxwell, 1956).

The lability of halogen attached to a hydroquinone ring was also observed with the iodinated compound (III). The D.A.R. of various tissues of a tumour bearing rat at 0·5 and 3 hours after injection of 10 mg. (III) is shown in Table II. The radioactivity of the thyroid is not given separately as it was found to contain no more radioactivity than the surrounding tissue. Again, the D.A.R. of the stomach was so high that dehalogenation was suspected and confirmed by paper

FIG. 3.—Radioactivity along a paper chromatogram of rat urine after injection of 5 mg. of tetrasodium 2 - methyl - 3 - $^{82}$Br - bromo - 1 : 4 - naphthohydroquinone diphosphate, showing positions occupied by bromide ion and by the injected compound (II).

Full curve : 0–2 hours.
Broken curve : 2–6 hours.
Ordinate : Radioactivity in counts per minute.
Abscissa : Distance from starting line (arbitrary units).

FIG. 4.—Paper electrophoresis of stomach contents of a rat 6 hours after intravenous injection of 10 mg. tetrasodium 2 - methyl - 3 - $^{82}$Br - bromo - 1 : 4 - naphthohydroquinone diphosphate showing position occupied by bromide ion.

Ordinate : Radioactivity in counts per minute.
Abscissa : Distance from starting line (arbitrary units).

FIG. 5.—Differential absorption ratio of organs of the rat after intravenous injection of 0·5 mg. (10 μC) of ammonium $^{82}$Br-bromide.
1, Stomach ; 2, lung ; 3, tumour ; 4, kidney ; 5, testis ;6, small intestine ; 7, liver ; 8, muscle.
Ordinate : Differential absorption ratio.
Abscissa : Time in hours after injection.

TABLE II.—*Differential Absorption Ratios* ± *Standard Errors*

| | 0·5 hours | 3 hours |
|---|---|---|
| Tumour | 1·1±0·2 | 0·7±0·1 |
| Muscle | 0·45±0·1 | 0·3±0·05 |
| Testis | 0·55±0·15 | 0·50±0·05 |
| Liver | 0·80±0·05 | 0·50±0·03 |
| Spleen | 0·85±0·05 | 0·40±0·05 |
| Kidney | 2·5±0·5 | 1·35±0·05 |
| Stomach + contents | 8·5±1·0 | 8·0±0·5 |
| | Percentage of Injected Dose | |
| Blood | 11·0±1·0 | 5·0±0·5 |
| Urine | 17·0±3·0 | — |

electrophoresis both of the stomach contents and of urine collected during the first 3 hours (Fig. 6 and 7).

From these data, we may conclude that the stomach contents and urine of rats receiving the iodo compound (III) contain large amounts of iodide ion and it is of interest to compare the resulting distribution with previous reports of the study of injected iodide. In the mammalian body, the distribution of injected iodide depends on the amount given. A high D.A.R. is shown by thyroid only if very small amounts are administered, and high uptake is then also found in the region of the pituitary (Phillips, 1955). The distrubution of larger doses is said to resemble that of chloride (Wallace and Brodie, 1937), but unlike chloride, the stomach concentrates iodide in the gastric juice relative to plasma (Leblond, 1948 ; Goldsmith, Stevens and Schiff, 1950). The distribution of 139γ of iodine in sodium $^{131}$I-iodide as reported by Stevens, Stewart, Quinlin and Meiken

(1949) is very similar to the distribution shown in Table II, especially at the 0·5 hour period. This confirms that even half an hour after injection, much of the label derived from compound (III) is present as free iodide ion ; at this dose

FIG. 6.—The distribution of radioactivity after paper electrophoresis of the contents of stomach of a rat 3 hours after injection of 10 mg. 2 : 3 - dimethyl - 5 : 6 - di$^{131}$I - iodobenzo-hydroquinone diphosphate (III) showing the positions occupied by iodide ion and by the injected compound.
Ordinate : Radioactivity in counts per minute.
Abscissa : Distance from starting line (arbitrary units).

FIG. 7.—The distribution of radioactivity obtained on paper electrophoresis of rat urine collected for 3 hours after injection of 10 mg. 2 : 3 - dimethyl - 5 : 6 - di$^{131}$I - iodo - 1 : 4 - benzohydroquinone diphosphate (III).
Ordinate : Radioactivity in counts per minute.
Abscissa : Distance from starting line (arbitrary units).

level (10 mg. (III) corresponding to 4 mg. iodine) thyroid would not be expected to have a higher D.A.R. than the surrounding muscle.

The results of these studies indicate the limitations of labelling a potential radiosensitizer of the hydroquinone diphosphate type with halogen in the hydroquinone ring.

SUMMARY

1. The distribution of radioactivity among the organs of rats bearing a Walker carcinoma following injections of 2-methyl-3-$^{82}$Br-bromo-1 : 4-naphthohydroquinone diphosphate, and of 2 : 3-dimethyl-5 : 6-di$^{131}$I-iodo-1 : 4-benzohydroquinone diphosphate has been studied.

2. Results show clearly that both compounds accumulate in tumour tissue to a greater extent than in other organs or muscle, but that the halogen atoms are quickly removed from the hydroquinone ring. Therefore, except for short periods after injection, the distribution of radioactivity is that of bromide or iodide ions respectively, with consequent high activity in the stomach contents.

3. The urine of the animals contains large amounts of the respective halide ion.

The authors wish to thank Professor J. S. Mitchell, F.R.S. for his helpful interest and Mr. E. A. King for his assistance with the animal experiments. They are also grateful to Dr. P. G. Jensen, of the Finsen Laboratories, for suggesting the use of the double counter. One of us (D.R.M.) is indebted to the Medical Research Council for a grant.

REFERENCES

Andrews, K. J. M., Marrian, D. H. and Maxwell, D. R.—(1956) *J. chem. Soc.*, 1844.
Davenport, H. W. and Fisher, R. B.—(1940) *Amer. J. Physiol.*, **131**, 165.
Friedmann, E., Marrian, D. H. and Simon-Reuss, I.—(1952) *Biochem. biophys. Acta*, **8**, 680.
Goldsmith, R. E., Stevens, C. D. and Schiff, L.—(1950) *J. Lab. clin. Med.*, **35**, 497.
Leblond, C. P.—(1948) *Advanc biol. med. Phys.*, **1**, 353.
Markham, R. and Smith, J. D.—(1952) *Biochem. J.*, **52**, 552.
Marrian, D. H. and Maxwell, D. R.—(1956) *Brit. J. Cancer*, **10**, 575.
Mitchell, J. S.—(1955) 'Radiobiology Symposium' (Liege), 1954. London (Butterworth's Scientific Publications), p. 170.
Perlman, I., Morton, M. E. and Chaikoff, I. L.—(1941) *Amer. J. Physiol.*, **134**, 107.
Phillips, A. F.—(1955) *Brit. J. Radiol.*, **28**, 440.
Roche, J., Michel, O., Michel, R., Gorbman, A. and Lissitzky, S.—(1953) *Biochim. biophys. Acta*, **12**, 570.
Stevens, C. D., Stewart, P. H., Quinlin, P. M., and Meiken, M. A.—(1949) *Cancer Res.*, **9**, 488.
Wallace, G. B. and Brodie, B. B.—(1937) *J. Pharmacol.*, **61**, 397.